# Early ctDNA Dynamics Predict Response to Mosperafenib in *BRAF *V600-Mutant Metastatic Colorectal Cancer

**DOI:** 10.1158/2767-9764.CRC-26-0196

**Published:** 2026-06-18

**Authors:** Martha Liliana Serrano-Serrano, Christina Godfried Sie, Oliver Bechter, Giulia Pretelli, María Vieito, Elisa Fontana, Catherine H. Han, Eduardo Castañón Álvarez, Ignacio Matos, David J. Pinato, Irene Moreno, Rikke L. Eefsen, Ruth Plummer, Reinhard Dummer, Hans Prenen, Joseph Sutton, Elisa Cinato, Gabriel Schnetzler, Nino Keshelava, Andreas Roller

**Affiliations:** 1 Roche Pharma Research and Early Development, Roche Innovation Center Basel, Basel, Switzerland.; 2 University Hospitals Leuven, Leuven, Belgium.; 3 Vall d’Hebron University Hospital, Barcelona, Spain.; 4 Sarah Cannon Research Institute, London, United Kingdom.; 5 New Zealand Clinical Research, Auckland City Hospital, Auckland, New Zealand.; 6 Clinica Universidad de Navarra, Madrid, Spain.; 7 Imperial College London, Hammersmith Hospital, London, United Kingdom.; 8 University of Piemonte Orientale, Novara, Italy.; 9 START Madrid-Centro Integral Oncológico Clara Campal (CIOCC), Hospital Universitario HM, Madrid, Spain.; 10 Department of Oncology, Herlev Gentofte Hospital, Copenhagen, Denmark.; 11 Newcastle Hospitals NHS Foundation Trust, Newcastle University, Newcastle, United Kingdom.; 12 Universitätsspital Zürich, Zürich, Switzerland.; 13 University Hospital Antwerp, Edegem, Belgium.; 14 Roche Pharma Research and Early Development, Roche Products Ltd, Welwyn Garden City, United Kingdom.; 15 Roche Pharma Research and Early Development, Roche Innovation Center Zurich, Zurich, Switzerland.

## Abstract

The serine–threonine protein kinase B-RAF (BRAF) V600 mutation confers a poor prognosis in metastatic colorectal cancer (mCRC). Mosperafenib is a novel, paradox-breaking BRAF inhibitor (BRAFi). In this analysis of a phase I study (ISRCTN13713551), we evaluated circulating tumor DNA (ctDNA) as a prognostic and predictive biomarker for mosperafenib monotherapy in *BRAF *V600-mutant mCRC. We analyzed 49 biomarker-evaluable patients with mCRC (*BRAF *V600-mutant; 23 BRAFi-naive, and 26 BRAFi-experienced). Plasma samples were collected at baseline and longitudinally. ctDNA levels were quantified using tumor-naive and tumor-informed assays and correlated with RECIST 1.1 response and progression-free survival (PFS). Mutational profiles were assessed to identify resistance mechanisms. Low baseline ctDNA (≤10% tumor fraction) was prognostic for longer median PFS (mPFS; 284 vs. 59 days; HR = 0.32, *P* = 0.00051). Early ctDNA dynamics were highly predictive; a ≥75% ctDNA reduction at cycle 1 day 15 (C1D15; “molecular response”) correlated with longer mPFS (281 vs. 43 days, *P* < 0.0001). This molecular response occurred in all BRAFi-naive patients versus 48% of BRAFi-experienced patients. Preexisting MAPK pathway resistance mutations were prevalent and prognostic for poor outcomes in the BRAFi-experienced cohort (HR = 3.5, *P* = 0.003), whereas BRAFi-naive patients acquired these at progression. ctDNA is a powerful biomarker for mosperafenib-treated *BRAF *V600-mutated mCRC. Low baseline ctDNA is highly prognostic, whereas an early, deep molecular response at C1D15 predicts durable benefit. This response is largely confined to BRAFi-naive patients, as preexisting MAPK pathway alterations in BRAFi-experienced patients correlate with lack of response. These findings support using early ctDNA dynamics as an efficacy endpoint in future trials.

**Significance:**

This study in *BRAF *V600-mutant mCRC treated with mosperafenib confirms ctDNA’s translational utility. Low baseline ctDNA associated with longer PFS and an early deep molecular response predicted durable clinical benefit, supporting its use as an early efficacy endpoint. In addition, ctDNA monitoring enables identification of resistance mechanisms.

## Introduction

Metastatic colorectal cancer (mCRC) remains a leading cause of cancer-related mortality worldwide. Mutations at valine 600 in the serine–threonine protein kinase B-RAF (BRAF) is observed in 8% to 12% of patients with mCRC and presents a negative prognostic factor in a microsatellite stable background regardless of disease stage (1). The mutation leads to constitutive activation of BRAF and downstream signal transduction in the MAPK pathway. Targeted therapies have been developed to block this oncogenic driver, but first-generation BRAF inhibitors (BRAFi) in monotherapy lead to recovery of phospho-ERK signaling and EGFR activation (2). This paradoxical reactivation of the MAPK pathway is clinically countered by combining BRAFi with EGFR inhibitors and chemotherapy (3–5). Mosperafenib is a novel, brain-penetrant BRAFi designed to overcome this paradox, showing promising activity in preclinical models (6). A phase I study evaluated the safety and tolerability of mosperafenib monotherapy through dose-escalation (7–9); here, we evaluate circulating tumor DNA (ctDNA) as a prognostic and pharmacodynamic biomarker.

Released by tumor cells into the bloodstream, ctDNA provides patient-specific information about the disease, capturing tumor heterogeneity and enabling real-time monitoring of response to treatment (10). Measurable ctDNA indicates residual disease, and whereas ctDNA levels show high interindividual variability, within-patient changes correlate with total tumor burden and can reflect responses to treatment or relapse (11). Thus, changes in ctDNA on treatment hold promise as a predictive PD biomarker. Although not currently validated as an early efficacy endpoint (12–14), regulatory agencies encourage analyzing ctDNA dynamics alongside imaging to assess antitumor activity and generate trial-specific evidence for the utility of early ctDNA response analysis (15). This evidence could inform subsequent trial parts, ultimately with the goal of bringing new therapies to patients sooner. We therefore examined ctDNA’s prognostic value at baseline, its therapy-driven dynamics, and its potential for noninvasive detection of resistance mutations.

It was previously demonstrated that patients with lower baseline *BRAF *V600E variant allele frequency (VAF) in blood had longer overall survival (OS) across treatment arms during a phase III trial of encorafenib (BRAFi) and cetuximab (EGFR antibody), irrespective of baseline covariates (16). Consequently, we assessed the prognostic value of both baseline *BRAF *V600-mutant VAF and ctDNA levels during mosperafenib monotherapy. Furthermore, to maximize the evaluable cohort for longitudinal monitoring, we assessed disease burden using tumor-informed (FoundationOneTracker, F1T; ref. 17) and tumor-uninformed (FoundationOne Liquid CDx, F1LCDx; refs. 18, 19) assays, depending on availability, to evaluate the predictive value of dynamic changes in ctDNA levels. Finally, we analyzed mutational profiles to detect preexisting and emerging resistance mechanisms using on-treatment and discontinuation plasma results derived from F1LCDx analysis.

## Materials and Methods

### Ethics statement

Study protocol and any amendments were approved by the local Institutional Review Board (IRB) at the participating sites. Patients provided written informed consent prior to any study-related procedures, and the study was performed in accordance with the Declaration of Helsinki and International Conference for Harmonization of Good Clinical Practice Guidelines. The study is registered on ISRCTN.com (ISRCTN13713551). The study was sponsored and funded by F. Hoffmann-La Roche.

### Study design and objectives

WP43295 was a first-in-human dose-escalation phase I study. Patients received mosperafenib monotherapy as either 25, 400, and 800 mg once daily; 800 or 1,600 mg twice daily; or 1,200 mg three times a day. The objectives of the study were to estimate the maximum tolerated dose and/or the recommended phase II dose, as well as safety and the tolerability, pharmacokinetics, and preliminary clinical activity of mosperafenib (8, 9). The exploratory endpoints discussed here evaluated whether genomic biomarkers correlate with RECIST 1.1 and progression-free survival (PFS). The study was performed in accordance with the Declaration of Helsinki and Good Clinical Practice guidelines, with local IRB approval and written informed consent from all patients (ISRCTN13713551).

### Study population and treatment

Eligible patients had histologically confirmed *BRAF* V600-mutant mCRC (per FDA-approved or CE-IVD test) progressing after one or more prior treatment regimens. A total of 92 patients were enrolled based on documented *BRAF *V600 mutation status, with 80 included in the safety and clinical outcome assessment and 51 in the colorectal cancer biomarker analysis subset (Supplementary Fig. S1). Of the 51 patients with colorectal cancer treated with mosperafenib monotherapy included in this analysis, 26 (51%) had received prior BRAFi treatment. Patients with stable/asymptomatic brain metastases were allowed. A complete list of eligibility criteria are described in the Appendix of Vieito and colleagues (7). Of the 51 patients with colorectal cancer, 49 were biomarker-evaluable [two were excluded as they were retrospectively assessed to be *BRAF *V600 wild-type (WT)]; 23 were BRAFi-naive (two of whom had prior cetuximab), and the 26 BRAFi-experienced patients were stratified as follows: prior BRAFi + cetuximab (*n* = 20), prior BRAFi but naive for cetuximab and MEKi (*n* = 3), and experienced for all three compounds (*n* = 3). Patients received mosperafenib monotherapy as either 25, 400, or 800 mg once daily; 800 or 1,600 mg twice daily; or 1,200 mg three times a day. Treatment duration was up to 28.6 months at time of data cutoff (median of 6.8 months).

### Sample collection and (ct)DNA analysis

Archival tissue samples were requested for retrospective confirmation of *BRAF* V600 mutation using F1LCDx (20). Longitudinal plasma samples were collected at cycle 1 day 1 (C1D1) before dose and at C1D15, C2D1, and C3D1 (cycle duration = 28 days) to assess ctDNA levels as early efficacy readout. Two ctDNA assays were used: the tumor-informed F1T (17) and the tumor-uninformed F1LCDx (18, 19). F1LCDx was used to determine baseline ctDNA tumor fraction (cTF, the proportion of ctDNA relative to total cell-free DNA in blood), *BRAF *V600 mutation, and profile preexisting and on-treatment resistance alterations. F1T was used for longitudinal quantification of mean tumor molecules (MTM/mL). The F1T assay was discontinued during the ongoing trial, and we decided to continue all longitudinal analyses with F1LCDx instead of F1T. Although F1LCDx has lower sensitivity, it captures mutation profiles, allowing us to detect potential resistance mechanisms on treatment and fill data missingness in the F1T dataset as it does not require baseline tissue.

### F1LCDx tissue mutation profiling and analysis

F1LCDx is an FDA-approved next-generation sequencing–based panel that is performed at a College of American Pathologists/Clinical Laboratory Improvement Amendments (CAP/CLIA)-certified laboratory using 27 to 1,000 ng DNA extracted from formalin-fixed, paraffin-embedded samples with at least 20% tumor nuclei content. It reports substitutions, indels, genomic rearrangements, and copy-number amplifications (CNA; amplifications and losses) in 324 cancer-related genes and genomic signatures, including tumor mutational burden (TMB) and microsatellite instability (MSI; ref. 18). The reported variants may be somatic or germline.

### FIT (tumor-informed ctDNA)

F1T assay was a tumor-informed ctDNA detection assay (17). A proprietary algorithm selected monitorable alterations that are highly likely somatic and not germline from F1CDx data. These were then quantified in plasma using a multiplex polymerase chain reaction assay adapted from the well-validated Signatera assay (21). The lowest reported values in this study were 0.001% mVAF and 0.0234 MTM/mL of plasma, respectively. The assay was discontinued during the course of the phase I study. In addition, we encountered issues with data missingness due to challenges with baseline data collection (available for only 32 patients, 65.3%) resulting from insufficient archival tissue or F1CDx failure. Therefore, imputation methods using a tumor-uninformed assay were implemented (described below). Furthermore, to correct for a substantial number of tracked mutations that were consistently undetected, MTM/mL values were recalculated based only on detectable alterations.

### F1LCDx (tumor-agnostic ctDNA)

The FDA-approved F1LCDx targets 324 cancer-related genes and is performed at a CAP/CLIA-certified laboratory using cell-free DNA extracted from plasma (18). The assay detects substitutions, indels, genomic rearrangements, and CNAs (amplifications and losses). The pipeline filters benign variants and reports variants of unknown significance as well as those with known or likely pathogenic effect based on an FMI internal curated knowledge database. Our analysis was focused on the variants reported as known or likely pathogenic, these may be somatic or germline variants. Genomic signatures, including bTMB, MSI, and cTF are also evaluated using proprietary algorithms. cTF is estimated based on aneuploidy or, when undetectable, from somatic alterations (19). The lowest reported value of cTF in this study was 0.19%, and the maximum 78%.

Associations between gene alterations in baseline and progression samples, where available, and survival outcomes were analyzed to explore the potential mechanisms of resistance to mosperafenib.

### MTM/mL missing values imputation

We performed regression analysis on 31 patients which had both F1T and F1LCDx readouts at baseline. As correlation between MTM/mL (F1T) and cTF (F1LCDx) was high (R = 0.93; *P* value = 1.5e−13; Supplementary Fig. S2A), we were able to compensate the high data missingness rate observed in the F1T dataset by converting cTF to MTM/mL, where necessary, using a linear regression model for imputation: ctDNA (MTM/mL) = β0 + β1 × (cTF), in which β0 is the intercept and β1 is the slope derived from the regression analysis. As an additional check, we converted cTF into MTM/mL using the following formula:

$$\frac{\left(\text{cfDNA}\;\text{extracted}\;\text{mass}\;\text{in}\;\text{ng}\right)\;\times \;\left(\text{cTF}\right)\;\times \;\left(1000\;\text{pg}/\text{ng}\right)}{\left(3.3\;\text{pg}\right)\;\times \;\left(\text{volume}\;\text{of}\;\text{plasma}\;\text{in}\;\text{mL}\right)}$$

Linear regression to MTM/mL determined by F1T on the same samples confirmed the validity of the conversion model (Supplementary Fig. S2B). A third regression on the conversion of VAF, plus plasma volume and DNA input into MTM/mL, showed good correlation with the linear regression (Supplementary Fig. S2B and S2C). In addition, unscheduled or discontinuation samples taken within 36 days after treatment were matched to the closest corresponding visit and used to complete early time series if necessary. This allowed for the assembly of a complete longitudinal dataset of early time points for 46 patients.

### Statistical considerations

Associations between baseline clinical variables and between those and ctDNA parameters were measured using a linear model for numeric, and McFadden pseudo R square for categorical variables (22). We examined the association between PFS and multiple clinical and molecular variables by comparing groups using a Cox regression proportional hazard model, with results reported as hazard ratios (HR), log rank test, and 95% confidence intervals (CI). Time to endpoints were summarized using Kaplan–Meier estimates along with the two-sided 95% Greenwood’s calculation. Median PFS (mPFS) durations (in months) were summarized along with 95% CIs in each treatment arm or biomarker subgroup. To mitigate potential immortality bias (23), a landmark analysis was applied to the hazard-based models that included on-treatment times. This approach includes only patients who were still at risk and the covariate was known at a prespecified time point. For continuous biomarkers derived from ctDNA without a defined cut-point, a maximal log-rank statistic (maxstat) approach was considered to estimate the predictor value that maximizes the difference in survival between the groups (24). All statistical analyses and most visualizations were done using R v 4.4.1 (RRID: SCR_001905).

## Results

### Clinical efficacy in the biomarker-evaluable population

Among the 49 biomarker-evaluable patients, clinical outcomes differed markedly based on prior treatment history. In the BRAFi-naive cohort (*n* = 23), mosperafenib monotherapy demonstrated meaningful clinical activity, with an objective response rate (ORR) of 26% and a mPFS of 9.6 months. In contrast, the BRAFi-experienced cohort (*n* = 26) showed limited efficacy, with an ORR of 7.7% and a mPFS of 3.6 months. These clinical results align with the broader phase I study population and provide the context for the subsequent biomarker analyses (7).

### Retrospective central *BRAF *V600 mutation analysis

We retrospectively obtained mutational profiles from archival tumor tissues of 44 patients by F1CDx and from blood at baseline of 50 patients by F1LCDx (Supplementary Fig. S1). *BRAF *V600 mutations were detected in 40 (91%) patients in tissue and in 43 (86%) patients in blood. Overall, the *BRAF *V600 mutation was confirmed in 47 of 51 patients with colorectal cancer by F1CDx and/or F1LCDx. Of the four patients with missing retrospective confirmation, two were retained in the subsequent analysis because false negativity could not be excluded (due to poor tissue quality and undetectable ctDNA). Patient enrollment criteria were locally confirmed *BRAF *V600 mutation status, and thus these two patients were retained in the analysis. However, the remaining two with high-quality data but unconfirmed *BRAF *V600 were excluded as they were considered *BRAF *V600 WT (19).

### Patient characteristics

**Figure 1.**
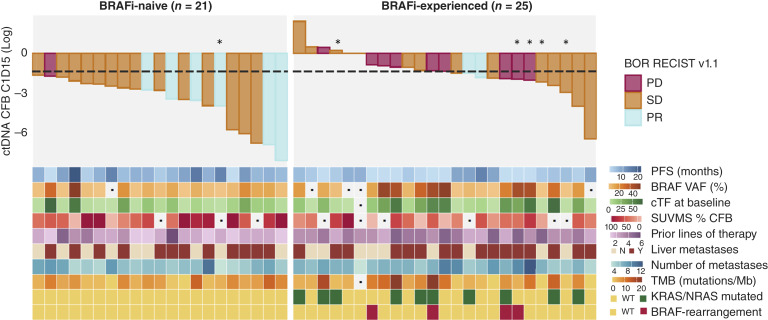
On-treatment ctDNA dynamics and associated baseline characteristics. Patient data are stratified by prior BRAFi treatment status: BRAFi-naive (*n* = 21) and BRAFi-experienced (*n* = 25). Top, waterfall plot illustrating the log-transformed CFB in ctDNA levels at C1D15. Each bar represents an individual patient, colored by their best overall response (BOR) per RECIST 1.1. The dashed line indicates a 75% reduction from baseline, the threshold for molecular response. For six patients with unavailable C1D15 data, the earliest available subsequent time point was used (marked with asterisk). Bottom, heatmap displaying key baseline and on-treatment characteristics for each patient. Black dots indicate missing. Mb, Megabase. [Created in BioRender. Godfried Sie, C. (2026) https://BioRender.com/akqu1mr.]

The median age of the 49 biomarker-evaluable patients was 60 years (24 males and 25 females). Prior BRAFi treatment represents a critical differentiating factor, given the frequent emergence of resistance mutations in those previously exposed to BRAF and MEK inhibitors (16). The patients included in the biomarker analysis exhibited a balanced distribution of BRAFi-naive (*n* = 23) and BRAFi-experienced patients [*n* = 26, average time since the last BRAFi treatment = 85 days (48–167); Table 1]. Patients with liver metastases were well-distributed between the two treatment history groups (57% in naive vs. 65% in experienced; *P* = 0.7326). Nonsignificant differences were observed in the number of metastases. Baseline ctDNA levels (3.90% vs. 16%, Wilcoxon *P* = 0.2) and baseline *BRAF *V600 VAF (3.53% and 15.85%, Wilcoxon *P* = 0.070) between BRAFi-naive and BRAFi-experienced patients show a notable trend that may reflect the more aggressive biology or higher disease burden typically seen in patients who have progressed on prior lines of therapy. The limited number of patients precludes a statistically significant difference but should be explored further in later studies. BRAFi-naive patients demonstrated a significantly deeper ctDNA reduction from baseline at all early on-treatment time points and derived greater benefit from the treatment compared with BRAFi-experienced patients (Wilcoxon rank test *P* < 0.001; Table 1; Fig. 1).

**Table 1.** Population characteristics and imbalances separated by prior BRAFi treatment.

CRC population characteristics		
Characteristic	Naive *N* = 23^a^	Experienced *N* = 26^a^
CBOR		
PD	1 (4.3%)	9 (35%)
SD	16 (70%)	15 (58%)
PR	6 (26%)	2 (7.7%)
Prior_CPI		
No	21 (91%)	23 (88%)
Yes	2 (8.7%)	3 (12%)
Number of metastasis	4 (3–6)	5 (4–6)
Liver metastasis		
No	10 (43%)	9 (35%)
Yes	13 (57%)	17 (65%)
SUV-max CFB at C1D15	−47.39 (−100 to −34.38)	−35.18 (−51.72 to −13.26)
(No assay/data available)	4	6
Baseline cTF % (F1LCDx)	3.90 (1.30–17)	16 (0.79–36)
(No assay/data available)	0	1
Baseline MTM/mL log (F1T)	4.11 (3.15–5.95)	5.28 (1.37–7.19)
(No assay/data available)	8	9
Baseline *BRAF *V600E % VAF	3.53 (1.81–10.48)	15.85 (2.65–44.93)
(No assay/data available)	2	4
ctDNA CFB at C1D15^b^	−2.81 (−4.01 to −2.33)	−1.38 (−1.96 to −0.89)
(No assay/data available)	2	1
ctDNA CFB at C2D1^b^	−3.68 (−4.76 to −2.33)	−0.82 (−2.04 to 0.16)
(No assay/data available)	6	8
ctDNA CFB at C3D1^b^	−3.83 (−5.33 to −2.10)	−0.91 (−2.24 to 0)
(No assay/data available)	5	16
Mut_Mbp	5.06 (2.53–8.85)	5.06 (2.53–11.38)
(No assay/data available)	0	1

Summary of baseline demographic and disease characteristics for the 49 biomarker-evaluable patients. Data are presented for the total population and stratified by prior treatment status (BRAFi-naive vs. BRAFi-experienced).

Abbreviations: CBOR, confirmed best overall response; CPI, checkpoint inhibitor; CRC, colorectal cancer; Mut_Mbp, mutations per megabase; SUV-max CFB, maximum standardized uptake value change from baseline.

a *n* (%); Median (Q1, Q3).

b ctDNA CFB is expressed in log difference.

### ctDNA levels at baseline and treatment response

**Figure 2.**
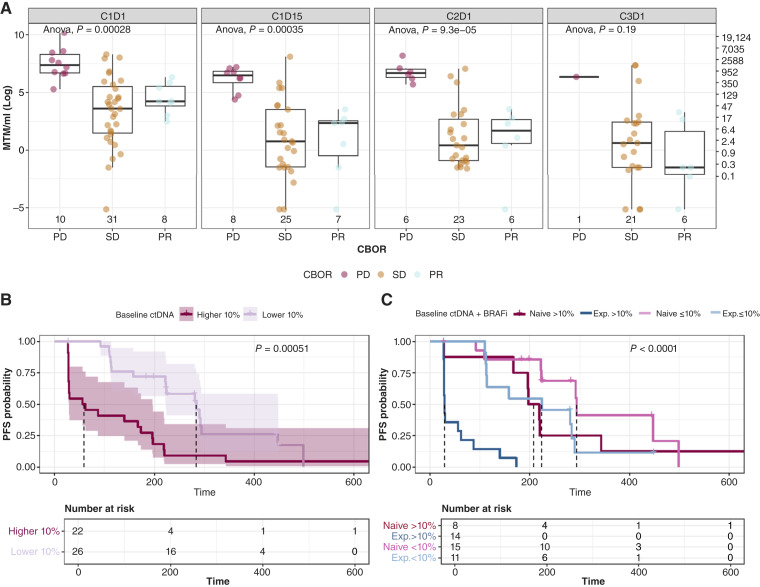
Baseline ctDNA as a prognostic factor in mCRC. **A,** Association of response (CBOR by RECIST 1.1) with ctDNA values (MTM/mL) at different cycles. Boxplots illustrating ctDNA levels—measured as MTM/mL—stratified by CBOR according to RECIST 1.1. PD patients exhibited significantly higher baseline and early on-treatment ctDNA levels compared with those with SD or PR. CBOR, confirmed best overall response. **B,** Kaplan–Meier analysis of PFS by baseline cTF (*n* = 48). PFS stratified by high (>10%) vs. low (≤10%) baseline cTF. A low baseline cTF was highly prognostic for a longer median PFS (284 vs. 59 days; HR = 0.32, *P* = 0.00051). **C,** Same analysis, further stratified by prior BRAFi treatment status. PFS analysis demonstrates that whereas prior BRAFi history affects survival, low baseline ctDNA remains a favorable prognostic signal across both treatment cohorts. *P* values in **A** were calculated via ANOVA; *P* values in **B** and **C** were determined by the log-rank test. Dashed lines in survival plots indicate mPFS. [Created in BioRender. Godfried Sie, C. (2026) https://BioRender.com/gms7f9h.]

Baseline ctDNA levels were associated with treatment response, with progressive disease (PD) patients showing significantly higher MTM/mL values than patients with stable disease (SD) or partial response (PR; Fig. 2A). Additionally, low baseline ctDNA is a strong prognostic factor for significantly improved mPFS; every logit unit of increase in ctDNA increases the risk of progression by 20% (HR = 1.2; *P* = 0.018; Supplementary Fig. S3). When using prespecified cutoffs based on larger colorectal cancer cohort estimates (25), which match very closely the cutoff optimization for this population (Supplementary Fig. S4), patients with low baseline ctDNA levels (≤10% cTF) experienced longer PFS (mPFS = 284 days) than those with ctDNA above 10% (mPFS = 59 days; HR = 0.32; *P* < 0.00051; Fig. 2B).

We further tested the association between ctDNA levels at baseline and clinical factors in a correlation matrix (Supplementary Fig. S5), confirming factors already shown to be associated with ctDNA levels at baseline: best overall response, molecular response, and *BRAF *V600-mutant VAF (16). We show that, additionally, *KRAS*/*NRAS *mutation status significantly affects baseline cTF with mean TF_WT_ = 0.14 (−3.33 logit) and TF_*KRAS*/*NRAS*mut_ = 0.34 (−0.95 logit; Supplementary Fig. S6A, Wilcoxon *P* = 0.0085), an interesting observation we sought to evaluate further as potential resistance mechanism, see below. Although the effects of prior BRAFi treatment on cTF and *BRAF *V600 VAF or liver metastases on baseline cTF are not significant, these associations may be worth exploring in future studies with larger cohorts (Supplementary Fig. S6B–S6D). However, the combination of baseline cTF and prior BRAFi treatment significantly affects PFS (Fig. 2C; *P* < 0.0001), and on treatment MTM/mL ctDNA levels and change from baseline significantly differ between prior BRAFi treatment states (Supplementary Fig. S6E and S6F), hinting at less effective ctDNA reductions in the face of prior BRAFi experience.

### *BRAF *V600 VAF in plasma at baseline

*BRAF* V600 VAF at baseline was reported in 43 patients with detectable ctDNA levels and ranged from 0.12% to 58.8% VAF (median of 8.16%). Of the six patients with no *BRAF *V600 VAF at baseline, three had undetectable ctDNA, two had ctDNA detected but not quantifiable, and for one patient the assay failed. Similar to ctDNA, higher levels of baseline *BRAF *V600-mutant VAF were associated with shorter PFS (HR = 2.8 for VAF > median, *P* = 0.01, Supplementary Fig. S7A and S7B), consistent with findings in other studies targeting BRAF (16, 26). We infer that this mutation occurred early in the tumor evolution, as it is present at a high proportion relative to the respective ctDNA levels. Consequently, these two biomarkers exhibit a strong correlation and possess comparable prognostic value (Supplementary Fig. S8; R = 0.97; *P* < 2.2e−16).

### Clinical variables and ctDNA prognostic value

**Figure 3.**
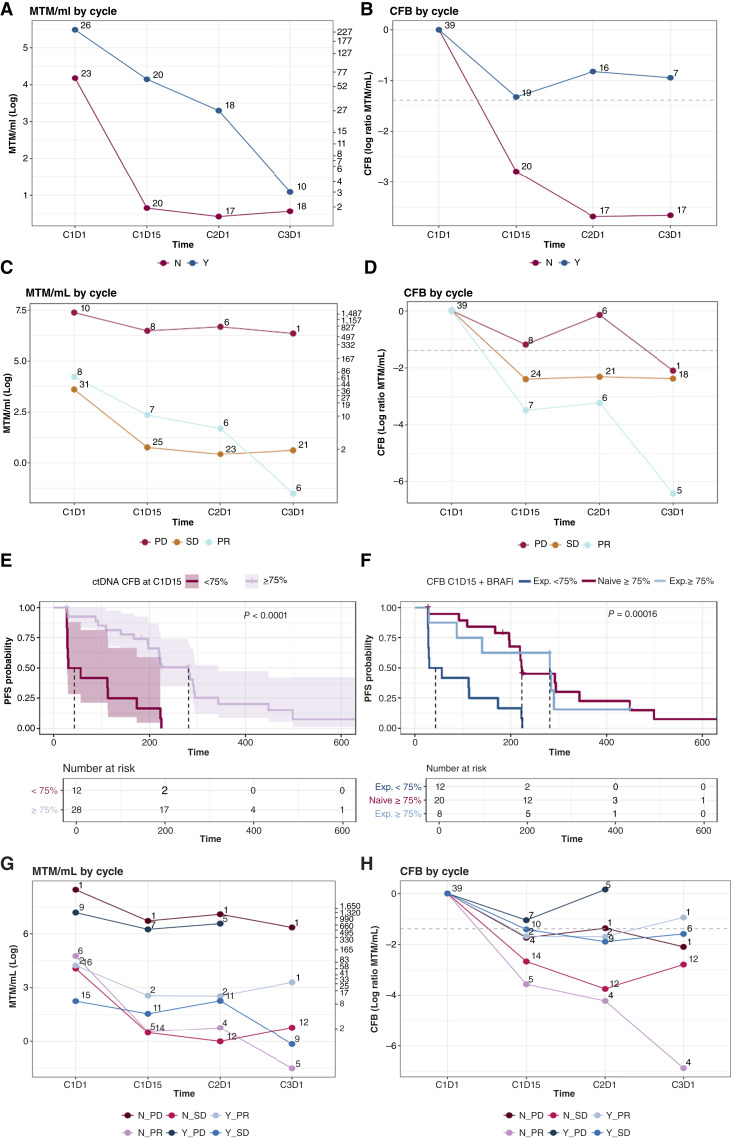
On-treatment ctDNA dynamics and response prediction. **A,** Median on-treatment ctDNA levels by treatment history: Log-transformed MTM/mL and change in log-transformed CFB. **B,** Across cycles, comparing BRAFi-naive (N) vs. BRAFi-experienced (Y) patients. BRAFi-naive (N) patients exhibited significantly deeper and more sustained ctDNA reductions compared with BRAFi-experienced (Y) patients. **C** and **D,** ctDNA dynamics by radiographic response: Median absolute levels (**C**) and CFB (**D**) stratified by best overall response. The dashed line in **D** indicates the −75% reduction threshold for molecular response. **E,** Kaplan–Meier analysis of molecular response: Patients achieving a −75% reduction in ctDNA by C1D15 showed significantly longer mPFS (281 vs. 43 days, *P* < 0.0001). **F,** Predictive value stratified by treatment history: Analysis showing that the durability of clinical benefit is linked to both the depth of molecular response and prior treatment status. **G** and **H,** Detailed trajectories by history and response: Combined stratification of absolute levels (**G**) and CFB (**H**) illustrating the distinct molecular patterns of naive partial responders (N_PR) vs. experienced progressors (Y_PD). CFB is expressed as a log ratio of MTM/mL. *P* values were calculated via the log-rank test. Dashed lines in **D** and **E** indicate mPFS. CFB is expressed in log difference. [Created in BioRender. Godfried Sie, C. (2026) https://BioRender.com/fnqpbyh.]

To identify the most prognostic parameters for *BRAF *V600-mutant mCRC patient PFS, we utilized a Cox regression model integrating clinical (PFS) and ctDNA variables. For the change from baseline (CFB) analysis, the C1D15 time point was chosen because of its high sampling frequency and its robust association with response (Fig. 2A). Best cutoff optimization showed that reduction of 75% resulted in the most significant association with response (Supplementary Fig. S9). All numerical parameters were treated as continuous predictors (Supplementary Table S1). Baseline and C1D15 ctDNA levels, as well as prior BRAFi treatment and *KRAS*/*NRAS* status, were the most prognostic factors. Further exploration suggests that baseline ctDNA and *BRAF *V600E VAF values are not different between naive and experienced patients (Supplementary Fig. S6C and S6D), but on-treatment ctDNA (C1D15) and CFB by C1D15 are (Fig. 3A and B; Supplementary Fig. S6E and S6F), and coupled with the presence of *KRAS*/*NRAS* mutations, suggest a higher residual tumor burden for the BRAFi-experienced population. As a consequence, the inclusion of on-treatment ctDNA (CFB at C1D15) information and prior BRAFi treatment in a multivariate Cox regression suggests a surrogate relationship between these two variables and PFS, rendering CFB a less significant independent prognostic factor (Supplementary Fig. S3).

### Longitudinal ctDNA and response prediction

We analyzed CFB in ctDNA levels using F1T and cTF values converted to MTM/mL for 46 patients (with samples collected at C1D1 and C1D15; if C1D15 was unavailable, C2D1 or C3D1 was used instead). For three patients only baseline values were available and were thus omitted from the longitudinal analysis. These changes correlated well with PFS and RECIST 1.1 criteria (Fig. 3C and D), with 80.5% of SD and PR patients showing more than 75% reductions in ctDNA (molecular responders) compared with only 40% of PD patients (Fig. 1). Molecular responders showed significantly longer PFS (mPFS = 281 days) compared with nonresponders (mPFS = 43 days, Fig. 3E and F). This effect may be confounded by prior BRAFi treatment, as BRAFi-naive patients generally exhibited a stronger molecular response with longer mPFS (Figs. 1, 3G, and H). All patients without prior BRAFi treatment and 48% (*n* = 12/25) of BRAFi-experienced patients showed at least a 75% reduction in ctDNA (Fig. 1; Supplementary Fig. S10A and S10B). Moreover, ctDNA dynamics for BRAFi-naive PR patients showed sustained strong reduction (at least until C3D1) in contrast to BRAFi-experienced PR patients who evolved similarly to SD patients (qualitative observation due to low sample size, Fig. 3G and H).

**Figure 4.**
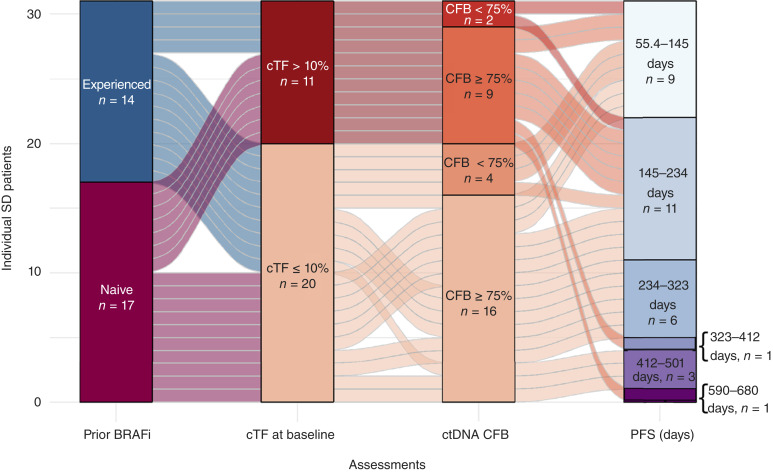
SD population dynamics (*n* = 31). Sankey diagram illustrating the flow of *N* = 31 patients who had SD at the C2D1 radiographic assessment. Patients are stratified by (i) prior BRAFi treatment status (naive vs. experienced), (ii) baseline cTF (>10% vs. ≤10%), (iii) on-treatment ctDNA molecular response at C1D15 (CFB ≥75% vs. <75%), and (iv) PFS in days. The diagram demonstrates that a deep molecular response (CFB ≥75%) was achieved by all BRAFi-naive patients and was typically associated with longer PFS. [Created in BioRender. Godfried Sie, C. (2026) https://BioRender.com/l0laxb0.]

ctDNA dynamics may offer valuable insights into the future progression of SD patients, potentially enabling earlier identification of those at risk. With this objective, we analyzed their ctDNA levels at baseline and changes at C1D15 to uncover molecular signals correlating with prolonged PFS. Figure 4 classifies SD patients as determined by first radiographic assessment at C2D1 (*N* = 31), stratifying them by prior BRAFi treatment status, baseline (cTF >10%) and changes in ctDNA (<75% reduction at C1D15), leading to their final PFS outcomes. Favorable ctDNA signals, ≤10% ctDNA at baseline and ≥75% reduction by C1D15, effectively identify patients that progress later with a predictive value of 0.7. We further incorporated this ctDNA SD classification within a Cox regression model, with HRs of 1.8 to 8.3, respectively, for the PFS endpoint of the SD categories at risk (Supplementary Fig. S11).

Given the dose-escalation nature of the study, we assessed the potential impact of dose intensity on molecular response. No significant association was observed between the administered dose of mosperafenib and the depth of ctDNA suppression at C1D15 (Supplementary Fig. S12), suggesting that molecular response was primarily driven by tumor biology rather than dose stratification within the biologically active range tested.

We also evaluated complete ctDNA clearance (100% reduction) as a potential endpoint. However, at the early C1D15 time point, undetectable ctDNA was a rare event (*n* = 6) and offered limited sensitivity compared with the ≥75% threshold. Furthermore, stronger ctDNA reduction by >95% did not better discriminate between PR and SD compared with the deep molecular response criteria (Supplementary Fig. S13).

### Resistance mechanisms

**Figure 5.**
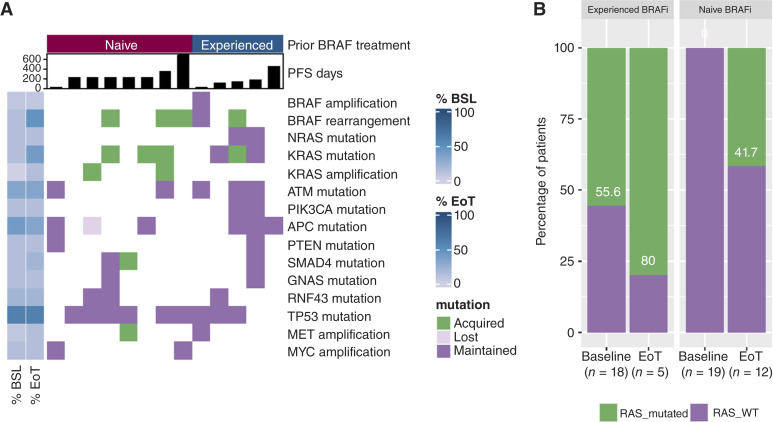
ONCOprint and tumor evolution. **A,** Oncoprint illustrating the landscape of mutated genes present in at least two patients at baseline and at discontinuation/progression, stratified by prior BRAFi treatment status (naive vs. experienced, top annotation) and PFS indicated as a bar graph below. The plot includes patients with ctDNA TF above the LOD at baseline and progression or with on-treatment data past cycle 3. The main grid indicates alteration status at progression: acquired (green), lost (pink), or maintained from baseline (purple). Left-hand annotations show the proportions of patients at baseline (% BSL) vs. at end-of-treatment (% eOT) exhibiting a mutation in that gene. Most notable are *de novo* acquisitions of *KRAS*/*NRAS *and *BRAF* rearrangement mutations. **B,** Prevalence of baseline *KRAS* or *NRAS* mutations (mutated vs. WT) in all samples with cTF above the LOD, stratified by prior BRAFi treatment status (Fisher test *P* = 0.0041, R-square association 30.1%). BSL, baseline; EoT, end-of-treatment. [Created in BioRender. Godfried Sie, C. (2026) https://BioRender.com/pp76eao.]

Of 37 patients with cTF above the limit of detection (LoD) for mutation detection (cTF >1%; ref. 19) at baseline, 15 (40.5%) had mutations in *APC *and 11 (29.7%) in ERK/MAPK pathway genes (Supplementary Table S2). Baseline mutations in *KRAS*, *NRAS*, and *BRAF* were found in 21.6%, 18.9%, and 13.5% of patients, respectively. Of 17 patients with cTF above the LoD at discontinuation or after cycle 9, the proportion of patients carrying MAPK pathway and activating *BRAF *mutations increased to 58.8% and 29.4%, respectively, whereas the proportion of *APC*-mutated tumors did not increase. Next, we looked at patients who had mutation profiles at both baseline and discontinuation available, separated by prior BRAFi treatment. Preexisting resistance mutations in the MAPK pathway were observed in baseline plasma samples of patients with prior BRAFi treatment (Fig. 5A). In contrast, among BRAFi-naive patients, such resistance mutations were detected at later time points or at discontinuation only. *RAS *mutations emerged in four of eight cases, and *BRAF *rearrangements evolved in three patients, two of which had novel cooccurring *KRAS *mutations or amplifications. Notably, similar rearrangements were also detected in a preclinical xenograft model treated with mosperafenib (27).

Patients with preexisting MAPK pathway activating mutations had higher baseline ctDNA values (Supplementary Fig. S6A) and shorter PFS than patients without (Supplementary Table S1, mPFS_*KRAS/NRAS* mut_ = 56 vs. mPFS_*KRAS/NRAS* WT_ = 223 days). This effect may reflect a potential resistance mechanism linked to prior BRAFi treatment, as naive patients were predominantly *KRAS*/*NRAS *WT, whereas those experienced were more likely to carry baseline mutations (Fisher test *P* = 0.0041, R-square association 30.1%, Fig. 5B), potentially driving higher baseline ctDNA values. PFS was similar for patients with acquired mutations compared with those with undetectable resistance mutations.

## Discussion

This translational study investigated the prognostic value of baseline ctDNA levels and the predictive power of early ctDNA dynamics in patients with *BRAF* V600-mutant mCRC tumor treated with mosperafenib monotherapy. Our findings establish ctDNA as a highly informative, real-time biomarker for patient stratification and early efficacy assessment in this specific patient population. Furthermore, the analysis of mutational profiles provided critical insights into both preexisting and acquired mechanisms of resistance to this novel BRAF paradox breaker inhibitor.

Mosperafenib treatment led to a clear, differential benefit based on prior treatment history. BRAFi-naive patients exhibited a significantly stronger molecular response and better clinical outcomes compared with BRAFi-experienced patients (Fig. 1; Table 1). The mPFS was substantially longer in the naive cohort, underlining the critical impact of prior exposure to MAPK pathway–targeting agents (8, 9).

The prognostic value of cTF at baseline was pronounced: patients with low baseline ctDNA (cTF ≤10%) experienced longer PFS (mPFS of 284 days) than those with high baseline levels (cTF >10%, mPFS of 59 days). This correlation reinforces the observation from other targeted therapy trials that overall tumor burden, as reflected by ctDNA, is a major determinant of clinical outcome, irrespective of the therapeutic agent (16, 28). Similarly, high *BRAF *V600-mutant VAF at baseline correlated with shorter PFS (HR = 2.8), which is consistent with its role as a predominant, often early, driver mutation in the primary tumor evolution.

Additionally, early changes in ctDNA levels were highly predictive of clinical response and PFS. A substantial reduction (≥75%) in ctDNA at C1D15 identified patients with significantly longer PFS (mPFS 281 days vs. 43 days), serving as an effective surrogate for antitumor activity (Fig. 3E). Although C1D15 ctDNA reduction proved a strong predictive factor in the univariate analysis, its prognostic significance was confounded by the inclusion of prior BRAFi treatment in the multivariate Cox model (Supplementary Fig. S3), suggesting a surrogate relationship: patients with prior BRAFi experience were less likely to achieve deep ctDNA reduction. These results underscore the prognostic value of on-treatment ctDNA dynamics (CFB at C1D15) in combination with prior BRAFi treatment history in patients with *BRAF *V600-mutant mCRC. Our efforts predicting the outcome of the SD population highlight the potential of early ctDNA dynamics as a valuable clinical biomarker for increased risk of progression, emphasizing its utility in complementing imaging assessments and potentially informing therapeutic interventions (29).

Whereas patients with SD and PR exhibited similar patterns of baseline TF and on-treatment dynamics, this overlap highlights the ability of ctDNA to detect drug activity independent of tumor shrinkage. Standard RECIST 1.1 criteria often fail to distinguish between SD patients with genuine therapeutic sensitivity and those with slow-growing, resistant disease. Our data suggest that a ≥75% reduction in ctDNA acts as a discriminator for “active” disease control, effectively identifying the subset of SD patients who derive durable survival benefit similar to partial responders.

Clinical benefit differences between patients in the naive and experienced subpopulations are strongly linked to the baseline landscape of MAPK pathway alterations. Preexisting resistance mechanisms, notably *KRAS *and *NRAS *mutations and *BRAF *rearrangements/amplifications, were significantly enriched at baseline in BRAFi-experienced patients (Figs. 1 and 5), which was also observed in preclinical PDX models (30). The Cox model confirmed that the *KRAS*/*NRAS *mutation status (HR = 3.5, *P* = 0.003) was the most significant negative prognostic factor for PFS, supporting that these concurrent mutations bypass the inhibitory effect of mosperafenib monotherapy (Supplementary Table S1).

Longitudinal analysis further elucidated the process of acquired resistance in naive patients. Whereas this naive cohort initially demonstrated deep molecular responses, *RAS* mutations emerged on treatment or at the time of discontinuation in four patients, and novel *BRAF* rearrangements evolved in three patients (Fig. 5A). These acquired alterations represent the clear mechanism of resistance, consistent with the expected selective pressure exerted by targeted therapy (31). Acquired *BRAF *rearrangements can generate fusion proteins that retain the kinase domain but lose regulatory regions, leading to constitutive, RAS-independent MAPK pathway activation. Similarly, BRAF kinase domain duplication promotes dimerization and sustained MAPK signaling (27, 32–34). Both mechanisms restore pathway activity despite targeted therapy, bypassing the inhibitory effect of mosperafenib and driving continued tumor growth (27). The dynamic monitoring capability of ctDNA facilitates real-time tumor genotyping and the identification of escape mechanisms, underscoring the need for combination or next-generation therapeutic strategies.

Our study has limitations inherent to a phase I design, including a small sample size and cohort heterogeneity. To address incomplete F1T data following assay discontinuation during the trial, we leveraged the high correlation between MTM/mL (F1T) and cTF (F1LCDx) for data imputation. Whereas this approach enabled more complete longitudinal analyses, combining tumor-informed (F1T) and tumor-uninformed (F1LCDx) assays introduces analytic complexity that may affect the reliability of predicted versus observed ctDNA trends. This limitation underscores a broader challenge for clinical implementation: the rapid evolution of ctDNA assay technology. Although standardization and validation protocols are essential (35–39), they cannot eliminate fundamental differences between tumor-informed and tumor-uninformed approaches. Rather, we may need to establish comparative validation frameworks—like the correlation-based approach used here—that document and quantify assay differences. This would allow clinicians to understand not just individual assay performance but how findings translate across platforms. In addition, although a single time point can be a strong response predictor, it may fail to capture tumors with resistant subclones that quickly escape the new treatment pressure, as exemplified by the ctDNA trajectories of individual patients (Supplementary Fig. S14A–S14F).

Beyond these technical considerations, a limitation is the clinical and molecular heterogeneity of the study population, including the intentional inclusion of both BRAFi-naive and BRAFi-experienced cohorts. As the clinical history directly correlates with the baseline prevalence of cooccurring *KRAS/NRAS* mutations, this collinearity between prior treatment experience and molecular resistance status warrants caution in interpreting the multivariate Cox model. This complex confounding highlights the necessity for larger, phase II studies to stratify for prior MAPK pathway inhibition, allowing for a cleaner assessment of predictive biomarkers like early ctDNA dynamics independent of preexisting resistance.

In conclusion, this study suggests the use of ctDNA as a powerful, noninvasive biomarker in *BRAF* V600-mutant mCRC patient population. Baseline ctDNA levels effectively stratify patients, and an early 75% reduction in ctDNA at C1D15 is highly predictive of favorable clinical outcome. Critically, longitudinal ctDNA monitoring provided real-time evidence of resistance evolution, linking acquired *RAS* and *BRAF* alterations to disease progression. These findings strongly support the integration of early ctDNA dynamics as an efficacy endpoint in future clinical trials of mosperafenib and similar agents, potentially accelerating go/no-go decisions or enabling timely alternative treatment decisions for patients with *BRAF *V600-mutant mCRC.

## Supplementary Material

Supplementary Figure S1 Patient analysis datasets

Supplementary Figure S2 ctDNA TF to MTM/ml regression model

Supplementary Figure S3 Multivariate Cox Regression for ctDNA variables

Supplementary Figure S4 Threshold optimization for cTF at baseline

Supplementary Figure S5 Correlation matrix between clinical and ctDNA variables

Supplementary Figure S6 Significant associations between clinical and ctDNA variables

Supplementary Figure S7 Cox regression forest plot for BRAF V600E allele frequency

Supplementary Figure S8 ctDNA TF and BRAF V600 VAF correlation

Supplementary Figure S9 Threshold optimization for CFB at C1D15

Supplementary Figure S10 ctDNA change from baseline separated by disease control and prior BRAFi treatment

Supplementary Figure S11 Cox regression model of ctDNA for SD classification

Supplementary Figure S12 Association between ctDNA change from baseline at C1D15 (log MTM/ml) and Mosperafenib dose

Supplementary Figure S13 Kaplan-Meier analysis of PFS stratified by molecular response

Supplementary Figure S14 Individual ctDNA traces as examples

Supplementary Table S1 Cox regression models for PFS with clinical and ctDNA variables

Supplementary Table S2 Mutations in specific pathways of interest at baseline and at discontinuation

## Data Availability

Anonymized records for individual patients across more than one data source external to Roche cannot, and should not, be linked due to a potential increase in the risk of patient reidentification. For up-to-date information on Roche’s Global Policy on the Sharing of Clinical Study Information and how to request access to related clinical study documents, please refer to https://go.roche.com/datasharing.
