# Supplementary material for: Early ctDNA Dynamics Predict Response to Mosperafenib in BRAF V600-Mutant Metastatic Colorectal Cancer
Source: Cancer Res Commun. 2026 Jun 18;6(6):1435–46. doi: 10.1158/2767-9764.CRC-26-0196 (PMC13276731; doi:10.1158/2767-9764.CRC-26-0196)
Supplement: Supplementary Figure S1 — Patient analysis datasets [file crc-26-0196_supplementary_figure_s1_suppsf1.pdf]

Supplementary Figure S1

Patient analysis datasets

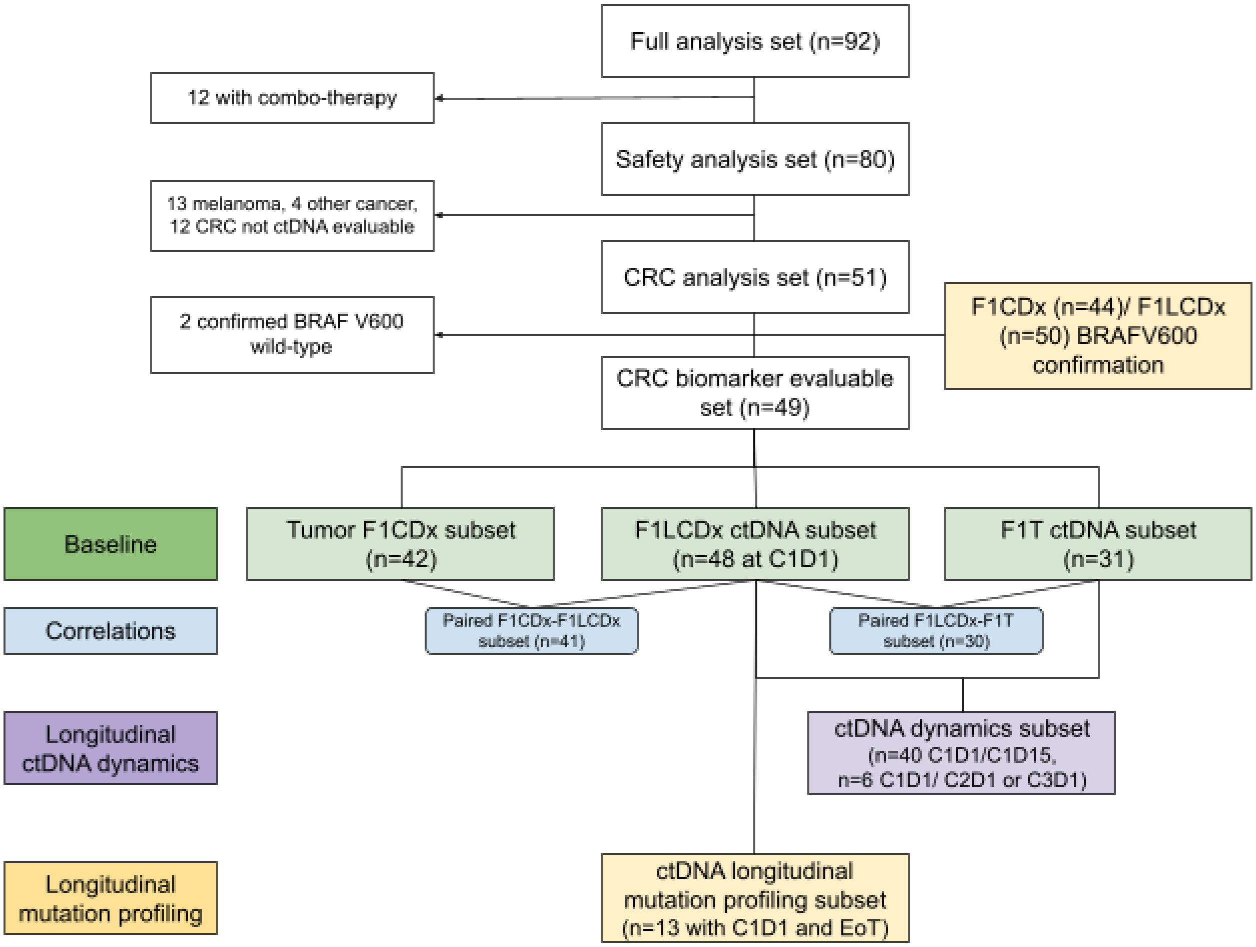

A total of 92 patients were enrolled in the study, of which 80 obtained monotherapy and were included in the safety analysis dataset. Of these, 13 were melanoma and 4 other cancers, and 12 CRC patients were from a formulation PK cohort and excluded from the biomarker analysis subset of 51. Retrospective confirmation of the BRAF V600 mutation status by F1CDx and/or F1LCDx, respectively, was negative for 2 patients. A total of 49 CRC biomarker evaluable patients remained. We performed F1CDx on archival tissue with valid results for 42, F1LCDx on plasma at baseline for 48; and 31 had F1T performed using personalized tracking of alterations

in plasma. We had paired F1CDx and F1LCDx subsets for 41 patients and paired F1LCDx and F1T ctDNA readouts for 30, which were used for the regression model (Supplementary Figure S1). The longitudinal ctDNA dynamics subset included 40 with paired C1D1/C1D15 and 6 with C1D1 plus an on-treatment datapoint of < 56 days from baseline. The final dataset included 46, of which the MTM/ml of 12 patients was imputed from F1LCDx cTF score in at least one timepoint. Finally, we analyzed the mutation profiles longitudinally of 13 patients
