# Supplementary material for: Early ctDNA Dynamics Predict Response to Mosperafenib in BRAF V600-Mutant Metastatic Colorectal Cancer
Source: Cancer Res Commun. 2026 Jun 18;6(6):1435–46. doi: 10.1158/2767-9764.CRC-26-0196 (PMC13276731; doi:10.1158/2767-9764.CRC-26-0196)
Supplement: Supplementary Figure S2 — ctDNA TF to MTM/ml regression model [file crc-26-0196_supplementary_figure_s2_suppsf2.pdf]

Supplementary Figure S2

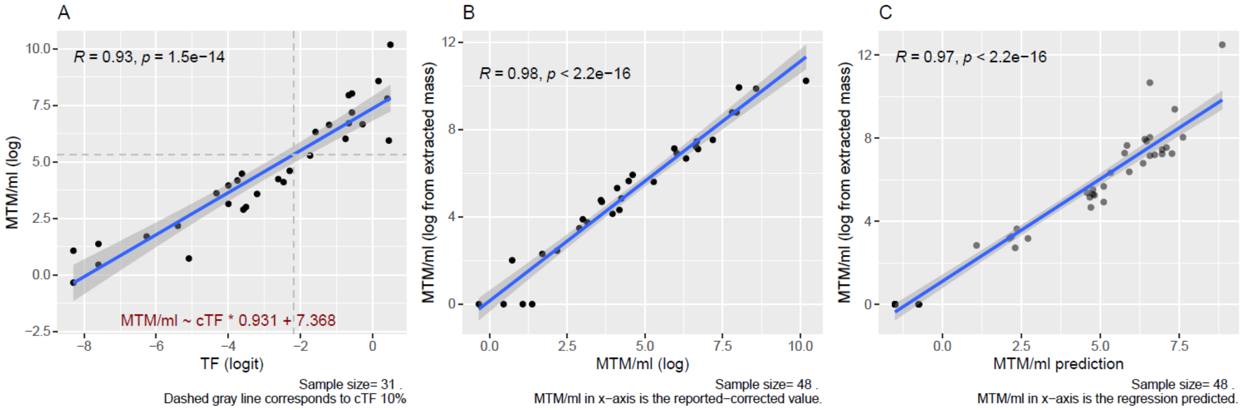

A) Regression model from patients with both assays at baseline (F1LCDx and F1T - with correction, see methods), B) Correlation between imputed MTM/ml values using the cfDNA extracted mass and F1T assay, C) Correlation between imputed MTM/ml values using the cfDNA extracted mass and MTM/ml prediction from the regression model (model formula in A was used to impute missing values from F1LCDx at baseline and on-treatment). The majority of longitudinal measurements were derived from F1T and thus the conversion was performed from cTF (readout from F1LCDx) to MTM/ml where necessary. See methods for further description.
