# Supplementary material for: Early ctDNA Dynamics Predict Response to Mosperafenib in BRAF V600-Mutant Metastatic Colorectal Cancer
Source: Cancer Res Commun. 2026 Jun 18;6(6):1435–46. doi: 10.1158/2767-9764.CRC-26-0196 (PMC13276731; doi:10.1158/2767-9764.CRC-26-0196)
Supplement: Supplementary Figure S3 — Multivariate Cox Regression for ctDNA variables [file crc-26-0196_supplementary_figure_s3_suppsf3.pdf]

Supplementary Figure S3

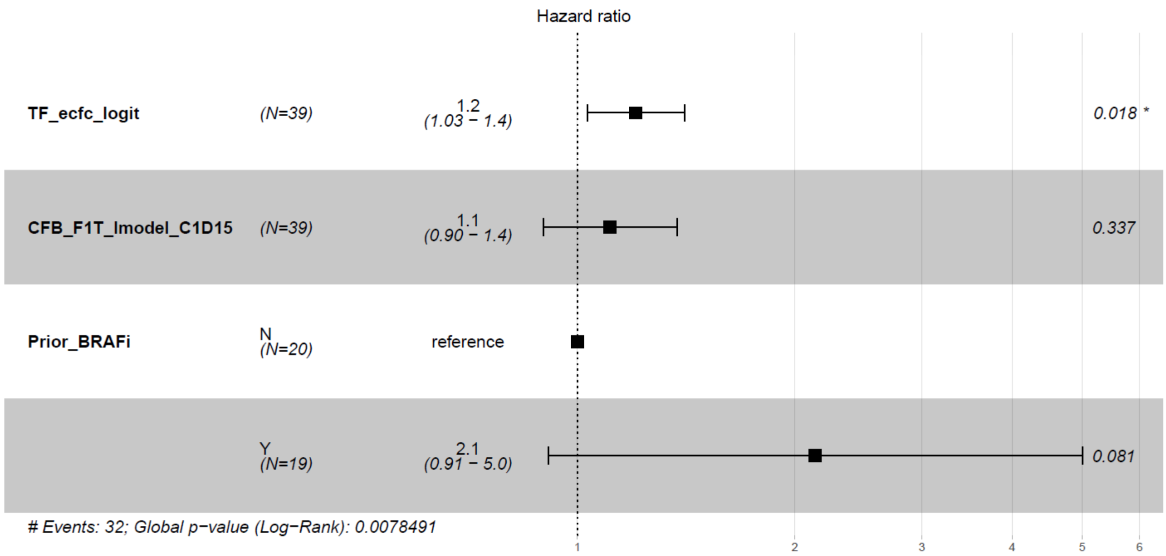

Multivariate Cox regression forest plot for ctDNA variables (n=39). Hazard ratio (HR) and 95% confidence interval are shown. For the continuous ctDNA variables (baseline TF and CFB), the Cox HR represents the risk ratio for every unit of change in the biomarker. However, for baseline TF notice there is a non-linear relationship between the transformed value (logit TF) value and the original TF [proportion 0-1].
