# Supplementary material for: Early ctDNA Dynamics Predict Response to Mosperafenib in BRAF V600-Mutant Metastatic Colorectal Cancer
Source: Cancer Res Commun. 2026 Jun 18;6(6):1435–46. doi: 10.1158/2767-9764.CRC-26-0196 (PMC13276731; doi:10.1158/2767-9764.CRC-26-0196)
Supplement: Supplementary Figure S4 — Threshold optimization for cTF at baseline [file crc-26-0196_supplementary_figure_s4_suppsf4.pdf]

Supplementary Figure S4

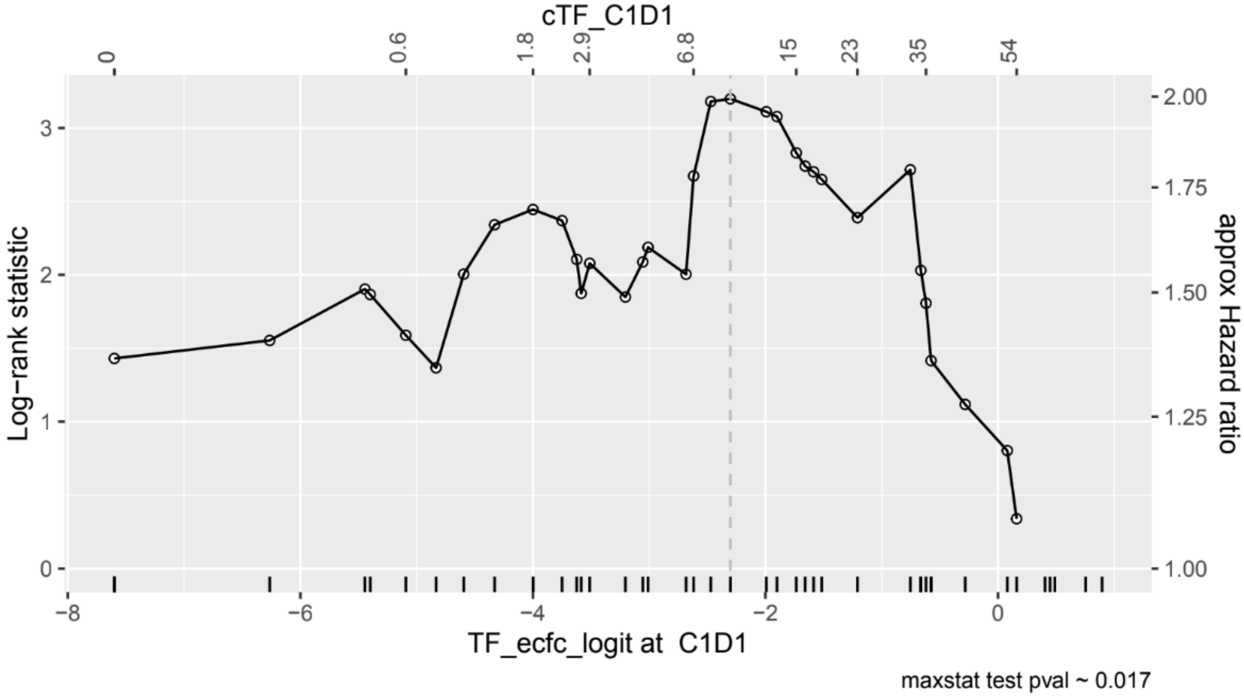

Threshold optimization for cTF at baseline to maximize progression-free survival (PFS) between patient subgroups. Maximal log-rank statistical value is considered as the best separation of groups (cTF ~10%). Baseline cTF threshold agrees with larger CRC cohort estimates.
