# Supplementary material for: Early ctDNA Dynamics Predict Response to Mosperafenib in BRAF V600-Mutant Metastatic Colorectal Cancer
Source: Cancer Res Commun. 2026 Jun 18;6(6):1435–46. doi: 10.1158/2767-9764.CRC-26-0196 (PMC13276731; doi:10.1158/2767-9764.CRC-26-0196)
Supplement: Supplementary Figure S5 — Correlation matrix between clinical and ctDNA variables [file crc-26-0196_supplementary_figure_s5_suppsf5.pdf]

Supplementary Figure S5

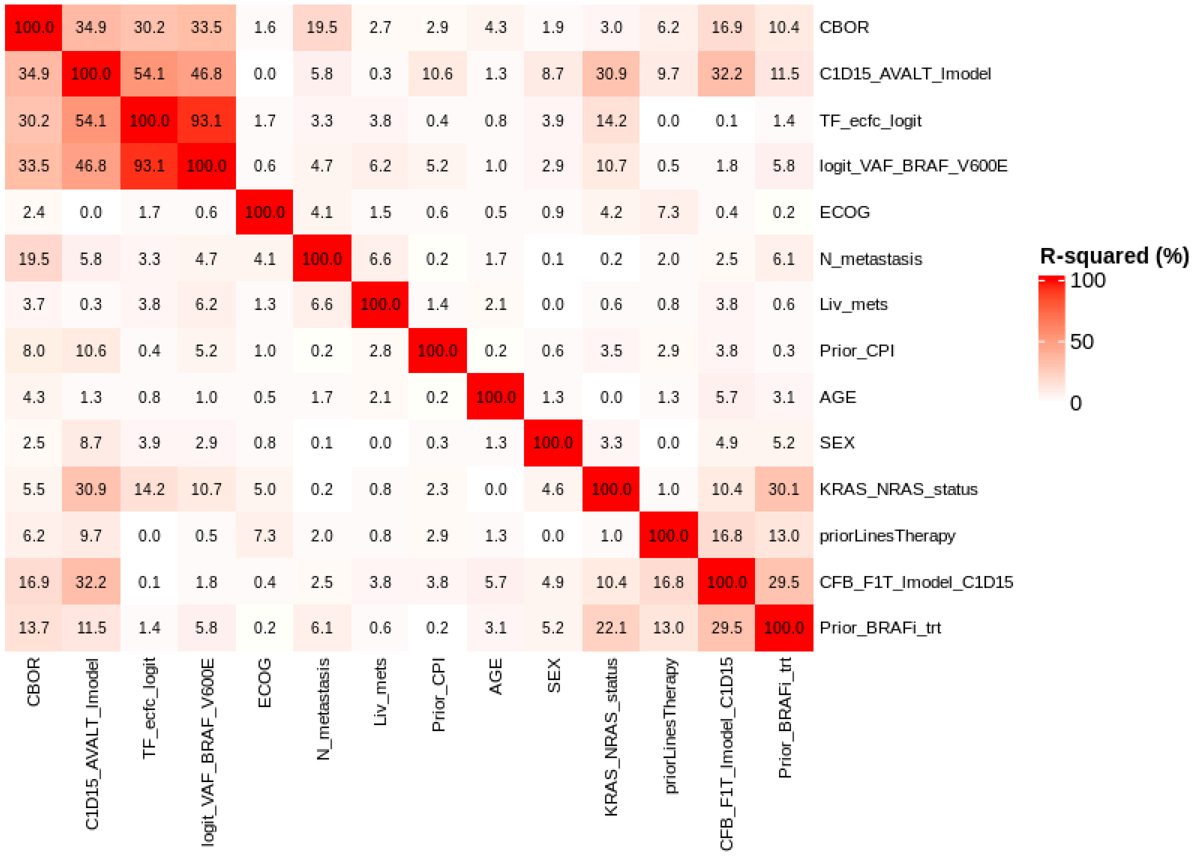

Correlation matrix amongst clinical variables and with ctDNA derived variables. R-square values indicate the level a variable in the row is influenced by the variables in the columns. Refer to figure S6 to see the direction of the trends.
