# Supplementary material for: Early ctDNA Dynamics Predict Response to Mosperafenib in BRAF V600-Mutant Metastatic Colorectal Cancer
Source: Cancer Res Commun. 2026 Jun 18;6(6):1435–46. doi: 10.1158/2767-9764.CRC-26-0196 (PMC13276731; doi:10.1158/2767-9764.CRC-26-0196)
Supplement: Supplementary Figure S6 — Significant associations between clinical and ctDNA variables [file crc-26-0196_supplementary_figure_s6_suppsf6.pdf]

Supplementary Figure S6

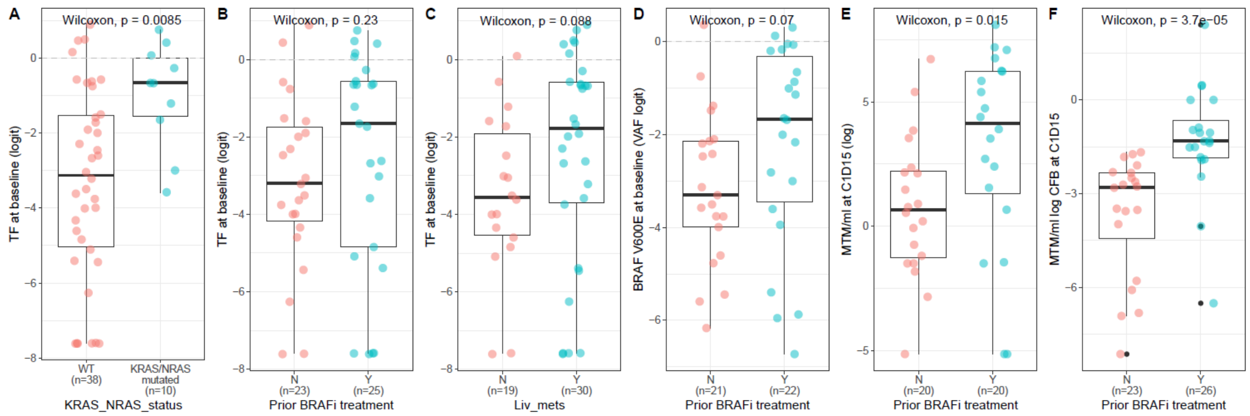

Significant associations between clinical and ctDNA variables (n=49 to n=39 depending on variable). By prior BRAFi treatment and KRAS/NRAS baseline status (A), ctDNA TF at baseline (B), liver metastatic state and ctDNA TF at baseline (C), BRAF V600E VAF (D), MTM/ml at C1D15 (E), MTM/ml CFB at C1D15 (F). Dashed gray line in A, B, C and D, corresponds to a 50% value (for TF or VAF).
