# Supplementary material for: Early ctDNA Dynamics Predict Response to Mosperafenib in BRAF V600-Mutant Metastatic Colorectal Cancer
Source: Cancer Res Commun. 2026 Jun 18;6(6):1435–46. doi: 10.1158/2767-9764.CRC-26-0196 (PMC13276731; doi:10.1158/2767-9764.CRC-26-0196)
Supplement: Supplementary Figure S7 — Cox regression forest plot for BRAF V600E allele frequency [file crc-26-0196_supplementary_figure_s7_suppsf7.pdf]

Supplementary Figure S7

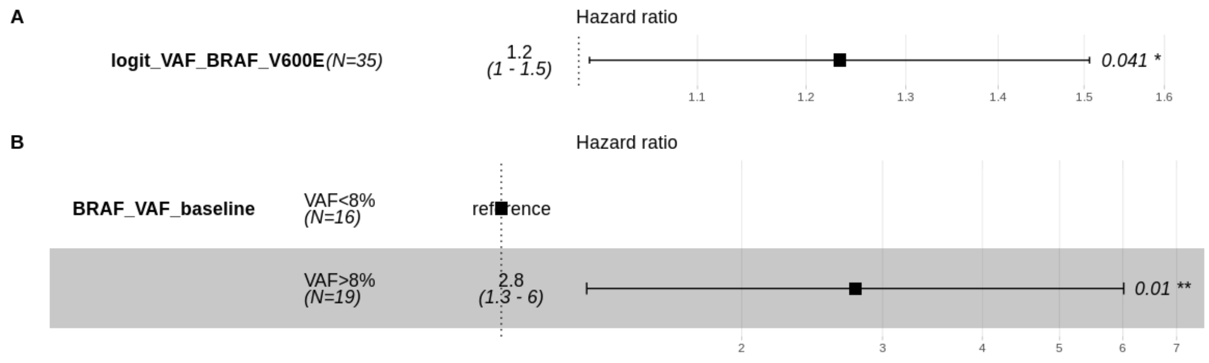

Cox regression forest plot for BRAF V600E allele frequency (n=35, excluding four patients with baseline cTF at limit of detection, compared to dataset in table S1). A) Linear association with logit scale VAF. Linear effect is very close to cTF (see table S1). B) split by population median (VAF = 8%).
