# Supplementary material for: Early ctDNA Dynamics Predict Response to Mosperafenib in BRAF V600-Mutant Metastatic Colorectal Cancer
Source: Cancer Res Commun. 2026 Jun 18;6(6):1435–46. doi: 10.1158/2767-9764.CRC-26-0196 (PMC13276731; doi:10.1158/2767-9764.CRC-26-0196)
Supplement: Supplementary Figure S8 — ctDNA TF and BRAF V600 VAF correlation [file crc-26-0196_supplementary_figure_s8_suppsf8.pdf]

Supplementary Figure S8

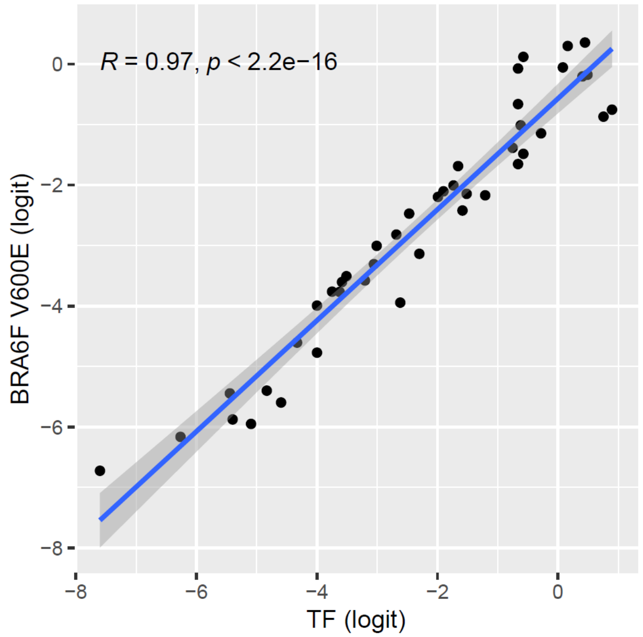

Association between cTF at baseline (logit scale) and BRAF V600 allele frequency (logit scale). Sample size 43. Patients with cTF below the limit of detection did not have a BRAF mutation identified (n=6).
