# Supplementary material for: Early ctDNA Dynamics Predict Response to Mosperafenib in BRAF V600-Mutant Metastatic Colorectal Cancer
Source: Cancer Res Commun. 2026 Jun 18;6(6):1435–46. doi: 10.1158/2767-9764.CRC-26-0196 (PMC13276731; doi:10.1158/2767-9764.CRC-26-0196)
Supplement: Supplementary Figure S9 — Threshold optimization for CFB at C1D15 [file crc-26-0196_supplementary_figure_s9_suppsf9.pdf]

Supplementary Figure S9

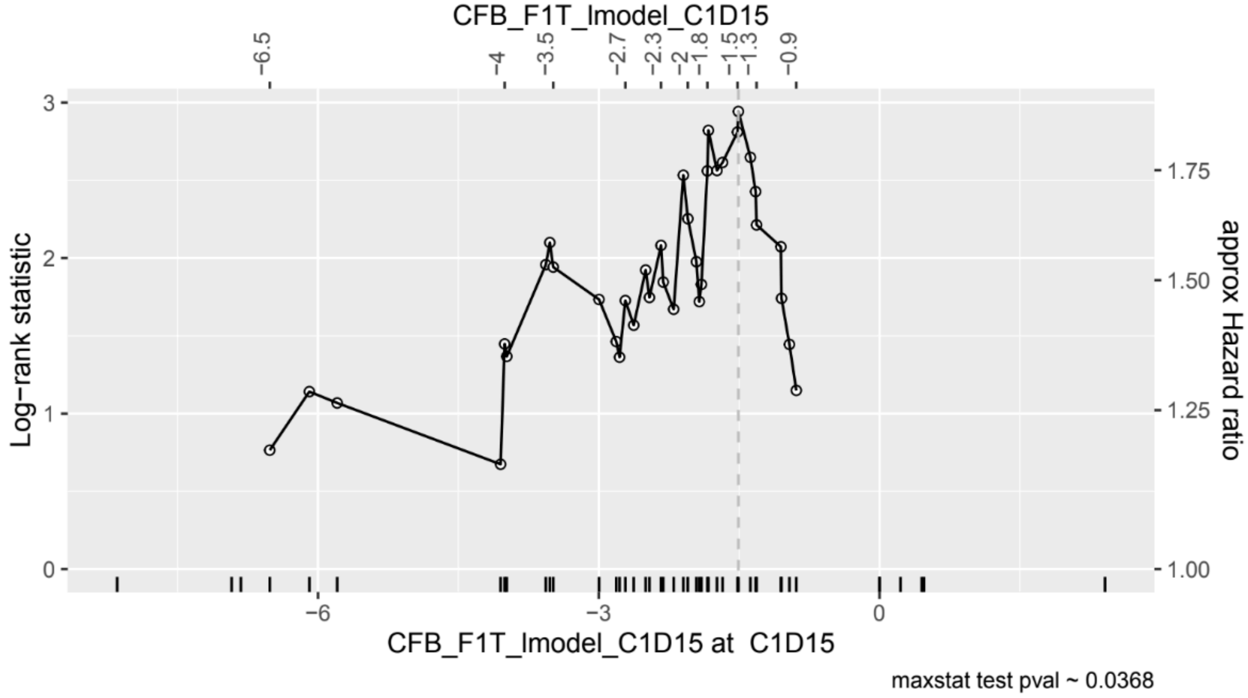

Threshold optimization for CFB at C1D15 to maximize progression-free survival (PFS) between patient subgroups. Maximal log-rank statistical value is considered as the best separation of groups (CFB ~75%, -1.507 in log ratio).
