# Supplementary material for: Early ctDNA Dynamics Predict Response to Mosperafenib in BRAF V600-Mutant Metastatic Colorectal Cancer
Source: Cancer Res Commun. 2026 Jun 18;6(6):1435–46. doi: 10.1158/2767-9764.CRC-26-0196 (PMC13276731; doi:10.1158/2767-9764.CRC-26-0196)
Supplement: Supplementary Figure S10 — ctDNA change from baseline separated by disease control and prior BRAFi treatment [file crc-26-0196_supplementary_figure_s10_suppsf10.pdf]

Supplementary Figure S10

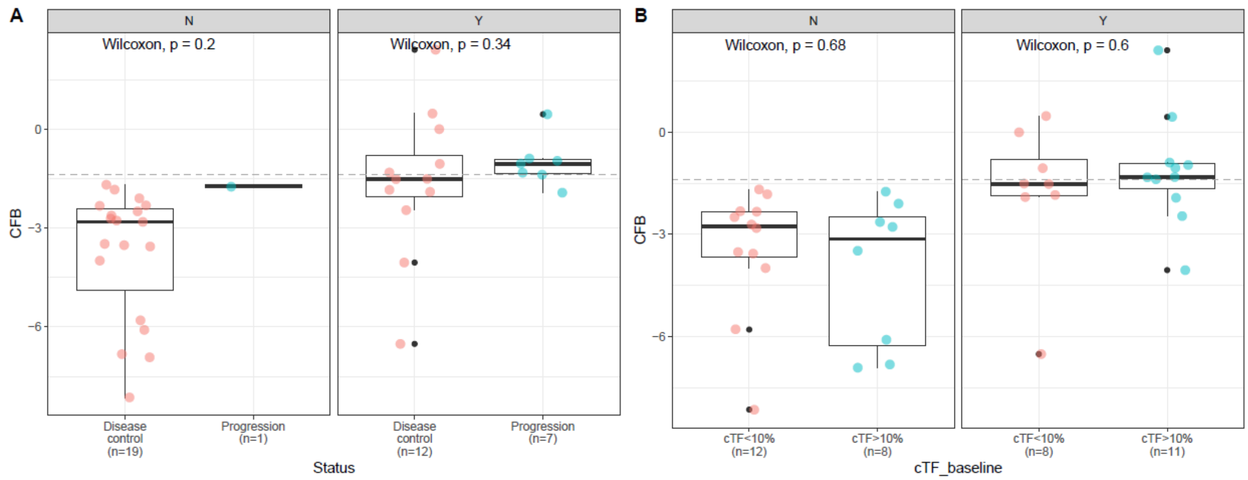

A) CFB at C1D15 separated by disease control and prior BRAFi treatment (N=39). B) CFB at C1D15 separated by prior BRAFi treatment and baseline ctDNA values. The threshold of -75% reduction is indicated by a dashed line.
