# Supplementary material for: Early ctDNA Dynamics Predict Response to Mosperafenib in BRAF V600-Mutant Metastatic Colorectal Cancer
Source: Cancer Res Commun. 2026 Jun 18;6(6):1435–46. doi: 10.1158/2767-9764.CRC-26-0196 (PMC13276731; doi:10.1158/2767-9764.CRC-26-0196)
Supplement: Supplementary Figure S11 — Cox regression model of ctDNA for SD classification [file crc-26-0196_supplementary_figure_s11_suppsf11.pdf]

Supplementary Figure S11

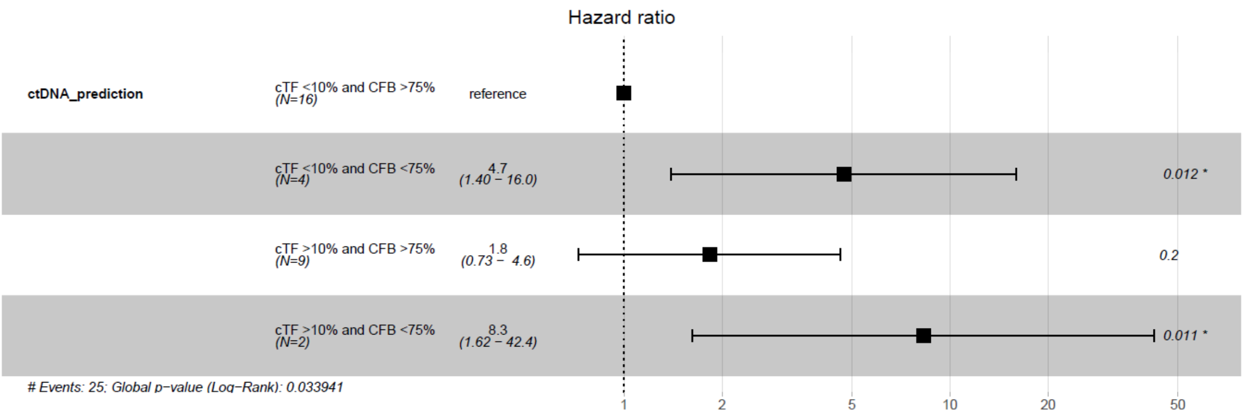

Forest plot for Cox regression model with combined ctDNA early signal classification.
