# Supplementary material for: Early ctDNA Dynamics Predict Response to Mosperafenib in BRAF V600-Mutant Metastatic Colorectal Cancer
Source: Cancer Res Commun. 2026 Jun 18;6(6):1435–46. doi: 10.1158/2767-9764.CRC-26-0196 (PMC13276731; doi:10.1158/2767-9764.CRC-26-0196)
Supplement: Supplementary Figure S12 — Association between ctDNA change from baseline at C1D15 (log MTM/ml) and Mosperafenib dose [file crc-26-0196_supplementary_figure_s12_suppsf12.pdf]

Supplementary Figure S12

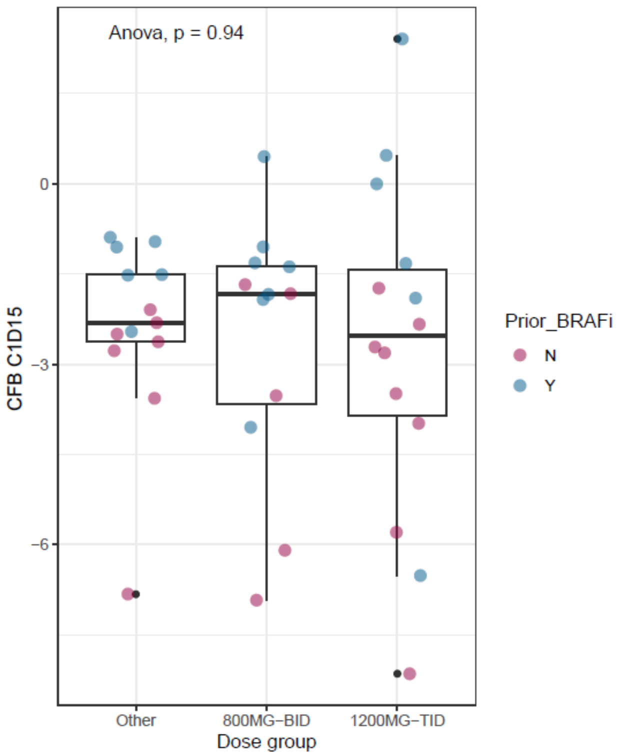

Association between ctDNA change from baseline at C1D15 (log MTM/ml) and Mosperafenib dose. Doses were simplified in 800 mg BID, 1200 mg TID or other (which includes any dose lower than 800mg BID). Anova test p.value = 0.94. Points are coloured by prior BRAF inhibitor status
