# Supplementary material for: Early ctDNA Dynamics Predict Response to Mosperafenib in BRAF V600-Mutant Metastatic Colorectal Cancer
Source: Cancer Res Commun. 2026 Jun 18;6(6):1435–46. doi: 10.1158/2767-9764.CRC-26-0196 (PMC13276731; doi:10.1158/2767-9764.CRC-26-0196)
Supplement: Supplementary Figure S13 — Kaplan-Meier analysis of PFS stratified by molecular response [file crc-26-0196_supplementary_figure_s13_suppsf13.pdf]

Supplementary Figure S13

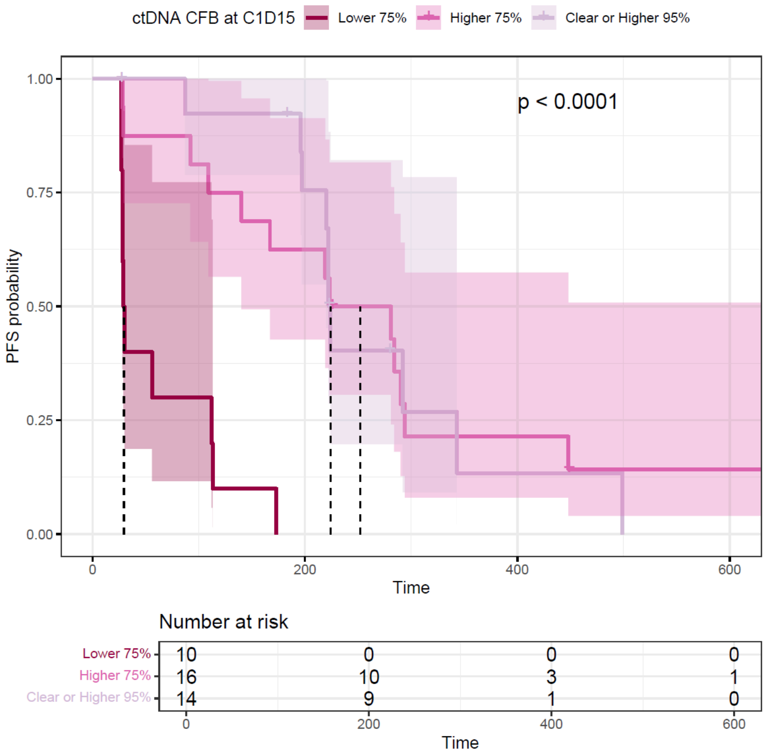

Kaplan-Meier analysis of PFS stratified by molecular response, defined as a <75% reduction (CFB <75%), ≥75% reduction (CFB ≥75%) and >95% reduction (CFB >95%) or clearance at C1D15 in ctDNA levels from baseline.. Dashed lines indicate median PFS. BRAFi, BRAF inhibitor; C1D15, Cycle 1 Day 15; CFB, change from baseline; ctDNA, circulating tumor DNA; MTM/ml, PFS, progression-free survival.
