# Supplementary material for: Early ctDNA Dynamics Predict Response to Mosperafenib in BRAF V600-Mutant Metastatic Colorectal Cancer
Source: Cancer Res Commun. 2026 Jun 18;6(6):1435–46. doi: 10.1158/2767-9764.CRC-26-0196 (PMC13276731; doi:10.1158/2767-9764.CRC-26-0196)
Supplement: Supplementary Figure S14 — Individual ctDNA traces as examples [file crc-26-0196_supplementary_figure_s14_suppsf14.pdf]

Supplementary Figure S14

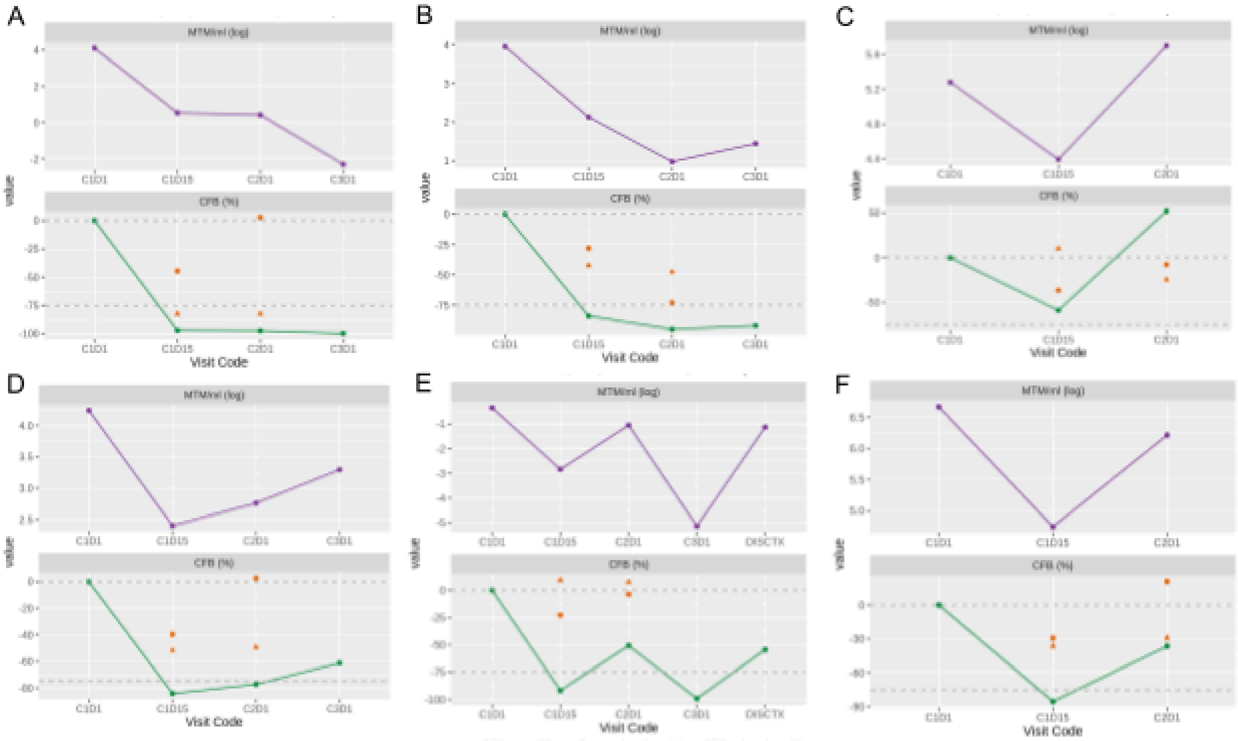

Individual ctDNA traces are shown for patients with PR (A, D), SD (B, E), and PD (C, F). Traces on the top row (A, B, C) are representative: PR (A) and SD (B) patients show > -75% reduction by C1D15; PRs become undetectable within the first three cycles, while SDs remain mostly detectable (B); PD patients typically fail to reach the 75% reduction by C1D15 and levels rebound quickly. Traces on the bottom row (D, E, F) are atypical: PR (D) show early ctDNA rebound with continued clinical benefit (PFS > 449); SD (E) showed irregular behavior due to very low, noisy ctDNA levels (<0.03%) and PD (f) showed an initial drop (-75% reduction by C1D15) but a quick rebound. Purple: absolute MTM/ml, log-transformed; green: relative CFB; red circles: TGLS and red triangles: SUVMS % CFB. CBOR: Confirmed Best Overall Response; PR: Partial Response; SD: Stable Disease; PD: Progressive Disease; C1D15: Cycle 1 Day 15; MTM/ml: Mean Tumor Molecules per milliliter; CFB: Change From Baseline; TGLS: Total Glycolytic Lesion Score; SUVMS: Standardized Uptake Value Maximum Score
