# Supplementary material for: Early ctDNA Dynamics Predict Response to Mosperafenib in BRAF V600-Mutant Metastatic Colorectal Cancer
Source: Cancer Res Commun. 2026 Jun 18;6(6):1435–46. doi: 10.1158/2767-9764.CRC-26-0196 (PMC13276731; doi:10.1158/2767-9764.CRC-26-0196)
Supplement: Supplementary Table S1 — Cox regression models for PFS with clinical and ctDNA variables [file crc-26-0196_supplementary_table_s1_suppst1.pdf]

Supplementary Table S1

Cox regression models for PFS with clinical and ctDNA variables (n=39). Median PFS (mPFS) is only calculated for covariates that have discrete states, not continuous. See figure 2 for discretization of baseline and CFB ctDNA.

Covariate (state)	HR	95% CI	mPFS (days)	Wald p.value
C1D15 MTM/ml predicted (log scale, N=39)	1.3	1.1 - 1.5	n.a.	*<0.001*
KRAS/NRAS status (mutated N=9)	3.5	1.5 - 8.1	WT= 223<br>mutated=56	*0.003*
Prior_BRAFi treatment (experienced N=19)	2.2	1.1 - 4.6	naive=223<br>exp.=112	*0.029*
Baseline cTF (logit scale, N=39)	1.2	1 - 1.4	n.a.	*0.044*
MTM/ml CFB at C1D15 (log scale, N=39)	1.2	0.99- 1.5	n.a.	0.063
Prior CPI (experienced N=3)	0.68	0.15 - 3.1	naive=219<br>exp.=28	0.619
Liver metastasis (present N=25)	1.0	0.51 - 2.1	absent=219<br>present=197	0.907

Univariate Cox proportional-hazards analysis for progression-free survival. Models evaluated key baseline clinical factors (e.g., prior BRAFi-treatment status) and ctDNA characteristics (e.g., tumor fraction, KRAS/NRAS co-mutation status), as well as on-treatment ctDNA dynamics. BRAFi, BRAF inhibitor; CFB, change from baseline; CI, confidence interval; CPI, checkpoint inhibitor; ctDNA, circulating tumor DNA; HR, hazard ratio; MTM/ml, Mean Tumor Molecules per milliliter; PFS, progression-free survival; cTF, composite tumor fraction.
