# Supplementary material for: Early ctDNA Dynamics Predict Response to Mosperafenib in BRAF V600-Mutant Metastatic Colorectal Cancer
Source: Cancer Res Commun. 2026 Jun 18;6(6):1435–46. doi: 10.1158/2767-9764.CRC-26-0196 (PMC13276731; doi:10.1158/2767-9764.CRC-26-0196)
Supplement: Supplementary Table S2 — Mutations in specific pathways of interest at baseline and at discontinuation [file crc-26-0196_supplementary_table_s2_suppst2.pdf]

Supplementary Table S2

	Baseline (n=37)	Discontinuation (n=17)
APC pathway	40.5% (n=15)	29.4% (n=5)
MAPK pathway	29.7% (n=11)	58.8% (n=10)
KRAS	21.6% (n=8)	52.9% (n=9)
NRAS	18.9% (n=7)	17.6% (n=3)
MAP2K1	2.7% (n=1)	nd
BRAF rearrangement	13.5% (n=5)	29.4% (n=5)
Deletions	8.1% (n=3)	23.5% (n=4)
Kinase domain duplication	2.7% (n=1)	nd
Amplification	2.7% (n=1)	5.9% (n=1)

Numbers of patients exhibiting mutations in specific pathways of interest at baseline (37 patients) and at discontinuation or at any time after cycle 9 (17 patients) with cTF above LoD of 1% for mutation detection. Note that some patients had co-mutations in KRAS and NRAS.
